# Effect of semaglutide on liver enzymes and markers of inflammation in subjects with type 2 diabetes and/or obesity

**DOI:** 10.1111/apt.15316

**Published:** 2019-06-10

**Authors:** Philip Newsome, Sven Francque, Stephen Harrison, Vlad Ratziu, Luc Van Gaal, Salvatore Calanna, Morten Hansen, Martin Linder, Arun Sanyal

**Affiliations:** ^1^ National Institute for Health Research Birmingham Biomedical Research Centre and Liver Unit at University Hospitals Birmingham NHS Foundation Trust Birmingham UK; ^2^ Centre for Liver & Gastrointestinal Research, Institute of Immunology and Immunotherapy University of Birmingham Birmingham UK; ^3^ Department of Gastroenterology and Hepatology Antwerp University Hospital Edegem, Antwerp Belgium; ^4^ Radcliffe Department of Medicine University of Oxford Oxford UK; ^5^ ICAN – Institute for Cardiometabolism and Nutrition Hôpital Pitié Salpêtrière, Sorbonne University Paris France; ^6^ Department of Endocrinology, Diabetology and Metabolism Antwerp University Hospital Edegem, Antwerp Belgium; ^7^ Novo Nordisk A/S Søborg Denmark; ^8^ Division of Gastroenterology, Hepatology and Nutrition, Department of Internal Medicine Virginia Commonwealth University Richmond Virginia

## Abstract

**Background:**

Obesity and type 2 diabetes are drivers of non‐alcoholic fatty liver disease (NAFLD). Glucagon‐like peptide‐1 analogues effectively treat obesity and type 2 diabetes and may offer potential for NAFLD treatment.

**Aim:**

To evaluate the effect of the glucagon‐like peptide‐1 analogue, semaglutide, on alanine aminotransferase (ALT) and high‐sensitivity C‐reactive protein (hsCRP) in subjects at risk of NAFLD.

**Methods:**

Data from a 104‐week cardiovascular outcomes trial in type 2 diabetes (semaglutide 0.5 or 1.0 mg/week) and a 52‐week weight management trial (semaglutide 0.05‐0.4 mg/day) were analysed. Treatment ratios vs placebo were estimated for ALT (both trials) and hsCRP (weight management trial only) using a mixed model for repeated measurements, with or without adjustment for change in body weight.

**Results:**

Elevated baseline ALT (men >30 IU/L; women >19 IU/L) was present in 52% (499/957) of weight management trial subjects. In this group with elevated ALT, end‐of‐treatment ALT reductions were 6%‐21% (*P*<0.05 for doses≥0.2 mg/day) and hsCRP reductions 25%‐43% vs placebo (*P *<* *0.05 for 0.2 and 0.4 mg/day). Normalisation of elevated baseline ALT occurred in 25%‐46% of weight management trial subjects, vs 18% on placebo. Elevated baseline ALT was present in 41% (1325/3268) of cardiovascular outcomes trial subjects. In this group with elevated ALT, no significant ALT reduction was noted at end‐of‐treatment for 0.5 mg/week, while a 9% reduction vs placebo was seen for 1.0 mg/week (*P* = 0.0024). Treatment ratios for changes in ALT and hsCRP were not statistically significant after adjustment for weight change.

**Conclusions:**

Semaglutide significantly reduced ALT and hsCRP in clinical trials in subjects with obesity and/or type 2 diabetes.

## 1 INTRODUCTION

Non‐alcoholic fatty liver disease (NAFLD) represents a spectrum of pathological conditions characterised by excessive hepatic fat deposition. It is currently the most common chronic liver disease in the world^1^, ^2^ and is estimated to affect as much as a quarter of the world's population.^3^ NAFLD is closely linked to insulin resistance and dyslipidaemia,^4^, ^5^ being highly prevalent among individuals with type 2 diabetes and/or obesity.^6^

A proportion of those with NAFLD will progress from steatosis to non‐alcoholic steatohepatitis (NASH), as characterised by inflammation and hepatocyte damage, which may in turn lead to the development of fibrosis, cirrhosis and hepatocellular carcinoma. Patients with NASH, particularly when accompanied by advanced fibrosis, have a greater mortality risk relative to the general population,^7^, ^8^, ^9^, ^10^ which in the main accrues from cardiovascular complications or malignancies^11^, ^12^ rather than end‐stage liver disease. However, NASH‐associated liver complications are currently the second leading indication for a liver transplant in the United States,^13^ both in the category of transplantation for cirrhosis^14^ and also for hepatocellular carcinoma.^15^

There are currently no licensed therapies for NASH, although the glucagon‐like peptide‐1 receptor agonists show promise due to their beneficial activity on glucose homeostasis and weight loss, as well as their anti‐inflammatory,^16^ lipid‐lowering^17^ and anti‐hypertensive effects.^18^ In addition, several glucagon‐like peptide‐1 receptor agonists have shown significant cardioprotective benefit for reducing major cardiac events in patients with type 2 diabetes at high cardiovascular risk.^19^, ^20^, ^21^

Liraglutide and semaglutide are two structurally related glucagon‐like peptide‐1 analogues indicated for the treatment of type 2 diabetes, both with established cardioprotective properties in these patients.^19^, ^20^ Liraglutide is also indicated for the treatment of obesity. Liraglutide has been observed to reduce elevated serum aminotransferases and hepatic steatosis in individuals with type 2 diabetes^22^ and, in a proof‐of‐concept randomised study, liraglutide treatment for 48 weeks resulted in histological resolution of biopsy‐proven NASH in 39% (9/23) of patients with or without type 2 diabetes. This compared with 9% [2/22] on placebo, with less worsening of fibrosis in the liraglutide group (9% [2/23] vs 36% [8/22]).^23^

Non‐alcoholic steatohepatitis is a common cause of elevated serum alanine aminotransferases (ALT),^24^ although the prevalence of NASH is also high among patients with type 2 diabetes who have normal ALT levels, particularly when accompanied by obesity.^25^ Serum C‐reactive protein is also predictive of NAFLD and has been linked to the presence and severity of underlying fibrosis.^26^ Although there are no currently approved medications for NASH, agents in early development which reduce hepatic fat content also display robust ALT reduction.^27^ Therefore, ALT changes can be considered a predictive marker for histological improvement. In the absence of hepatic histological data for glucagon‐like peptide‐1 receptor agonists, it is important to explore the effect of these drugs on ALT changes, in available datasets, to gain a better understanding of their potential benefit in NAFLD/NASH. Herein, we report the results of a post hoc analysis evaluating the effect of semaglutide on levels of ALT and C‐reactive protein in subjects enrolled in two clinical trials of semaglutide treatment for obesity or type 2 diabetes, two conditions related to NASH.

## 2 METHODS

### 2.1 Study designs

Data were drawn and analysed post hoc from two published clinical trials from the semaglutide development programme: a weight management trial in subjects with obesity without diabetes, and a cardiovascular outcomes trial in older individuals with type 2 diabetes and elevated cardiovascular risk. These two populations were dosed differently, with semaglutide given once daily for obesity and once weekly for diabetes in the cardiovascular outcomes trial.

### 2.2 Weight management trial (NCT02453711)

NCT02453711 was a phase 2, randomised, double‐blind, multinational, placebo‐ and active‐controlled dose‐finding trial of semaglutide in combination with both dietary and exercise counselling. The study is fully described elsewhere,^28^ but briefly, semaglutide was given once daily for 52 weeks at subcutaneous doses of 0.05, 0.1, 0.2, 0.3 or 0.4 mg to individuals with obesity of non‐endocrine origin (body mass index ≥30 kg/m^2^) without diabetes.

Semaglutide was initiated at the lowest dose of 0.05 mg/day and sequentially escalated to the next level every 4 weeks until reaching the final assigned dose. For doses of 0.3 and 0.4 mg/day, two additional exploratory groups were recruited with escalation every 2 weeks. The active comparator was liraglutide 3.0 mg, initiated at 0.6 mg and escalated to final dose by an additional 0.6 mg every week. For all active treatment groups (semaglutide or liraglutide), participants were randomised 6:1 to active drug or a matched placebo of identical dosing volume and escalation schedule, and all placebo groups were pooled for analysis. All subjects received hypocaloric dietary advice and individualised exercise counselling on a monthly basis.

The study enrolled and treated 957 subjects, 102‐103 per active group and 136 in the pooled placebo group. The primary endpoint was percentage weight change from baseline to week 52, estimated by analysis of covariance with missing data imputed from the placebo pool using a multiple imputation jump‐to‐reference approach. Overall, 81% (777/957) of subjects received the full 52 weeks of treatment, and week 52 weight data were also available for an additional 12% (115/957) of “retrieved” participants who returned for evaluation after early treatment discontinuation.

### 2.3 Cardiovascular outcomes trial (SUSTAIN‐6; NCT01720446)

SUSTAIN‐6 was a phase 3, randomised, double‐blind, multinational, placebo‐controlled trial of semaglutide given for the treatment of type 2 diabetes. Full details of this study are also published,^20^ but briefly, semaglutide was given once weekly at subcutaneous doses of 0.5 or 1.0 mg/week for 104 weeks to individuals at least 50 years of age with type 2 diabetes and a haemoglobin A_1c_ level ≥7%, at high risk for, or with a prior history of, cardiovascular events and/or who had chronic kidney disease. Semaglutide was initiated at 0.25 mg/week and escalated to final dose on a 4‐weekly schedule. For both semaglutide groups, randomisation was 1:1 between active drug and a matched placebo. Both the semaglutide and placebo groups were pooled for the primary analysis of overall treatment vs placebo, but were not pooled for analysis of secondary endpoints.

The study enrolled 3297 subjects, of whom data were available for 3232 (98%) at week 104. The primary endpoint was the occurrence of a major adverse cardiac event, consisting of cardiovascular‐related death or the first occurrence of a nonfatal myocardial infarction or stroke.

### 2.4 Analyses

For the weight management trial, baseline characteristics are shown for the full cohort, but in‐trial data are shown only for the placebo pool and the five semaglutide treatment groups on 4‐weekly escalation (0.05, 0.1, 0.2, 0.3 and 0.4 mg/day). Changes are not shown for the exploratory 2‐weekly escalation groups or liraglutide 3.0 mg, although the placebo pool includes subjects randomised to the matched placebos for these three groups. For the cardiovascular outcomes trial, baseline characteristics and in‐trial data are presented for all participants, and no groups were pooled for analysis.

The NAFLD Fibrosis Score^29^ and Fibrosis 4 Index^30^ were calculated at baseline for both trials. Results were classified as high, indeterminate or low, based on the risk of advanced fibrosis, using both global and age‐stratified thresholds based on published data.

Thresholds for high and low NAFLD Fibrosis Score were >0.676 and ≤−1.455, respectively. The negative predictive value for advanced fibrosis has been reported to be 88%‐93% for scores below the low cut‐off and positive predictive values of 82%‐90% for scores above the high cut‐off in a cohort of 733 biopsy‐confirmed NAFLD patients.^29^ Thresholds for high and low Fibrosis 4 Index were >3.25 and ≤1.45, respectively. The negative predictive value for advanced fibrosis has been reported to be 95% for scores below the low cut‐off and positive predictive values of 82% for scores above the high cut‐off in a cohort of patients with chronic hepatitis C infection.^31^ Subjects with NAFLD Fibrosis Score or Fibrosis 4 Index values between the low and high thresholds were considered to have an indeterminate result.

Since the predictive value of both the NAFLD Fibrosis Score and Fibrosis 4 Index is known to decline outside the age range 35‐65 years, age‐stratified thresholds for both markers were also applied according to the algorithm of McPherson et al.^32^ Subjects aged 35 years or less were not classified under either score. For the NAFLD Fibrosis Score, the global thresholds described above were applied to subjects aged 36‐64 years, while for those aged 65 years or more the high and low thresholds were set at >0.676 and <0.12, respectively. For the Fibrosis 4 Index, high and low scores of >2.67 and <1.3, respectively, were applied to all subjects aged 36‐64 years, while for those aged 65 years or more the thresholds were >2.67 and <2.0, respectively.

The presence of metabolic syndrome at baseline was assessed in both trials according to the criteria of the 2009 harmonised definition,^33^ using the European/North American thresholds for waist circumference. Metabolic syndrome was defined as three or more of: waist circumference ≥89 cm (women) or ≥102 cm (men); triglycerides ≥1.7 mmol/L; high‐density lipoprotein cholesterol <1.3 mmol/L (women) or <1.04mmol/L (men); systolic blood pressure ≥130 mm Hg and diastolic blood pressure ≥85 mm Hg; fasting plasma glucose ≥5.6 mmol/L.

ALT at baseline and during the trial was assessed centrally in both trials, and participants were classified as having high (>30 IU/L in males or >19 IU/L in females) or normal levels at baseline. These cut‐offs, based on a reference population at low risk of subclinical liver disease, were originally suggested by Prati et al.^34^

Changes in ALT from baseline were analysed by baseline ALT subgroup using a mixed model for repeated measurements with log‐transformed baseline ALT as the covariate, and with treatment, sex and either region (weight management trial) or stratification (cardiovascular outcomes trial; nine strata) as fixed factors. To explore to what extent changes in ALT were associated with weight loss, a second model was constructed that was adjusted for body weight change. This weight‐adjusted model used the same fixed factors and log‐transformed baseline ALT covariate as the unadjusted model, plus baseline body weight and change from baseline body weight as additional covariates. All covariates and factors for both models were nested within visit and subgroup. Treatment ratios vs placebo were estimated from the model at weeks 28 and 52 in the weight management trial, and weeks 30, 56 and 104 in the cardiovascular outcomes trial.

Changes in high‐sensitivity C‐reactive protein (hsCRP) from baseline were analysed by baseline ALT subgroup in the weight management trial only, as this parameter was not assayed in the cardiovascular outcomes trial, using the same weight‐adjusted and ‐unadjusted mixed‐model approach and with the same factors as for ALT, but with log‐transformed baseline hsCRP replacing ALT as a covariate. Additional weight‐unadjusted model analyses of changes in both ALT and hsCRP in the weight management trial were undertaken by baseline ALT subgroup in combination with either sex or age relative to the median (<47 years vs ≥47 years).

All analyses of ALT and hsCRP in the weight management trial used data collected during the trial irrespective of whether the subject was on trial medication. However, data on these parameters were not collected under the study protocol from those retrieved participants who discontinued drug but returned for week 52 weight assessment.

## 3 RESULTS

The primary results from both trials are fully described elsewhere.^20^, ^28^ Briefly, in the weight management trial the estimated weight changes on semaglutide were dose dependent and ranged from −6% to −14% of baseline in the 4‐weekly escalation groups, which was superior to placebo (−2%) at all doses and superior to liraglutide (−8%) at all semaglutide doses above 0.1 mg/day. In the cardiovascular outcomes trial, semaglutide treatment was associated with a hazard ratio of 0.74 (95% confidence interval: 0.58‐0.95) vs placebo for the major adverse cardiac event endpoint.

### 3.1 Baseline characteristics

Baseline characteristics in both studies are shown by baseline ALT subgroup in Table 1, and differed between the two studies. Compared with the weight management trial, the cardiovascular outcomes trial population with type 2 diabetes was older, and had lower body weight, lower cholesterol, more male subjects (61% vs 35%) and a higher proportion of subjects with metabolic syndrome (80% [2588/3252] vs 53% [502/953]). The proportion with elevated baseline ALT was greater in the weight management trial (52% [499/957] vs 41% [1325/3268]), and, although the gender balance in each ALT subgroup was similar in the weight management trial, disproportionately more women in the cardiovascular outcomes trial had elevated ALT than men (Table 1). Within each subgroup of normal or elevated ALT, the median ALT level was similar between the two trials. In both trials the proportion of subjects with metabolic syndrome was higher in the high ALT subgroup. The majority of participants in the weight management trial (65% [621/953] of those with data) had elevated baseline hsCRP (>3.0 mg/L).

**Table 1:** Baseline characteristics

Median (range) unless otherwise indicated	NCT02453711 (weight management trial)		SUSTAIN‐6 (cardiovascular outcomes trial)	
	High ALT^a^ (n = 499)	Normal ALT^a^ (n = 458)	High ALT^a^ (n = 1325)	Normal ALT^a^ (n = 1943)
Age (y)	48 (18‐76)	47 (19‐86)	63 (50‐88)	65 (50‐89)
Male, n (%)	187 (37.5)	151 (33.0)	600 (45.3)	1383 (71.2)
Weight (kg)	106.9 (70.5‐216.3)	107.8 (70.2‐243.7)	91.6 (46.7‐178.3)	88.8 (40.7‐216.8)
BMI (kg/m^2^)	37.4 (29.7‐77.1)	37.9 (29.7‐80.3)	33.0 (19.4‐61.4)	31.2 (17.6‐77.7)
Waist circumference (cm)	116.8 (82.2‐180.0)	114.8 (83.3‐187.0)	110.3 (73.7‐179.3)	107.7 (68.4‐173.7)
HbA_1c_ (%)	5.5 (4.3‐6.6)	5.5 (4.2‐7.0)	8.4 (6.0‐16.6)	8.3 (5.9‐17.9)
Total‐C (mmol/L)	5.2 (2.7‐9.7)	5.0 (2.6‐10.3)	4.4 (2.0‐14.6)	4.2 (1.7‐16.4)
LDL‐C (mmol/L)	3.2 (1.1‐6.2)	3.0 (0.8‐7.2)	2.2 (0.1‐10.2)	2.2 (0.3‐10.3)
HDL‐C (mmol/L)	1.2 (0.5‐2.4)	1.3 (0.7‐2.9)	1.1 (0.4‐3.5)	1.1 (0.4‐3.5)
Triglycerides (mmol/L)	1.6 (0.5‐11.9)	1.4 (0.4‐9.9)	2.0 (0.5‐38.0)	1.6 (0.1‐16.2)
ALT (IU/L)	34.0 (20.0‐313.0)	17.0 (3.0‐30.0)	35 (20‐580)	18 (5‐30)
AST (IU/L)	24.0 (12.0‐272.0)	16.0 (8.0‐62.0)	28 (13‐453)	18 (6‐75)
APRI	0.3 (0.1‐3.4)	0.2 (0.1‐0.9)	0.3 (0.1‐4.5)	0.2 (0.1‐3.4)
Systolic BP (mm Hg)	128.0 (94.0‐184.0)	125.0 (87.0‐176.0)	135.0 (74.0‐204.0)	135.0 (84.0‐203.0)
Diastolic BP (mm Hg)	81.0 (55.0‐119.0)	80.0 (55.0‐105.0)	79.0 (40.0‐107.0)	77.0 (44.0‐116.0)
hsCRP (mg/L) ≥3 mg/L, n/N (%)	4.1 (0.2‐42.2) 312/498 (62.7)	5.0 (0.2‐105.5) 309/455 (67.9)	ND	ND
FPG (mmol/L) ≥6.1 mmol/L, n/N (%)	5.4 (3.6‐12.0) 76/499 (15.2)	5.3 (4.2‐9.8) 57/457 (12.5)	9.9 (2.8‐26.8) 1226/1315 (93.2)	9.4 (2.5‐40.2) 1753/1928 (90.9)
Metabolic syndrome^b^, n/N (%)	293/497 (59.0)	209/456 (45.8)	1138/1319 (86.3)	1450/1933 (75.0)
NFS	–1.69 (–5.83; 3.18)	–1.33 (–5.16; 3.53)	–0.36 (–5,23; 3.07)	–0.22 (–6.71; 5.35)
FIB‐4	0.73 (0.14‐3.31)	0.69 (0.19‐2.52)	1.24 (0.38‐6.54)	1.14 (0.32‐14.96)

ALT, alanine aminotransferase; APRI, AST‐to‐platelet ratio index; AST, aspartate aminotransferase; BMI, body mass index; BP, blood pressure; C, cholesterol; FIB‐4, Fibrosis 4 Index; FPG, fasting plasma glucose; HbA_1c_, haemoglobin A_1c_; HDL, high‐density lipoprotein; hsCRP, high‐sensitivity C‐reactive protein; LDL, low‐density lipoprotein; ND, not determined; NFS, non‐alcoholic fatty liver disease Fibrosis Score.

a High ALT was classified as >30 IU/L in males and >19 IU/L in females.^34^

b Metabolic syndrome defined as three or more of: waist circumference ≥89 cm (women) or ≥102 cm (men); triglycerides ≥1.7 mmol/L; HDL‐C <1.3 mmol/L (women) or <1.04 mmol/L (men); systolic blood pressure ≥130 mm Hg and diastolic blood pressure ≥85 mm Hg; FPG ≥5.6 mmol/L.

**Figure 1:**
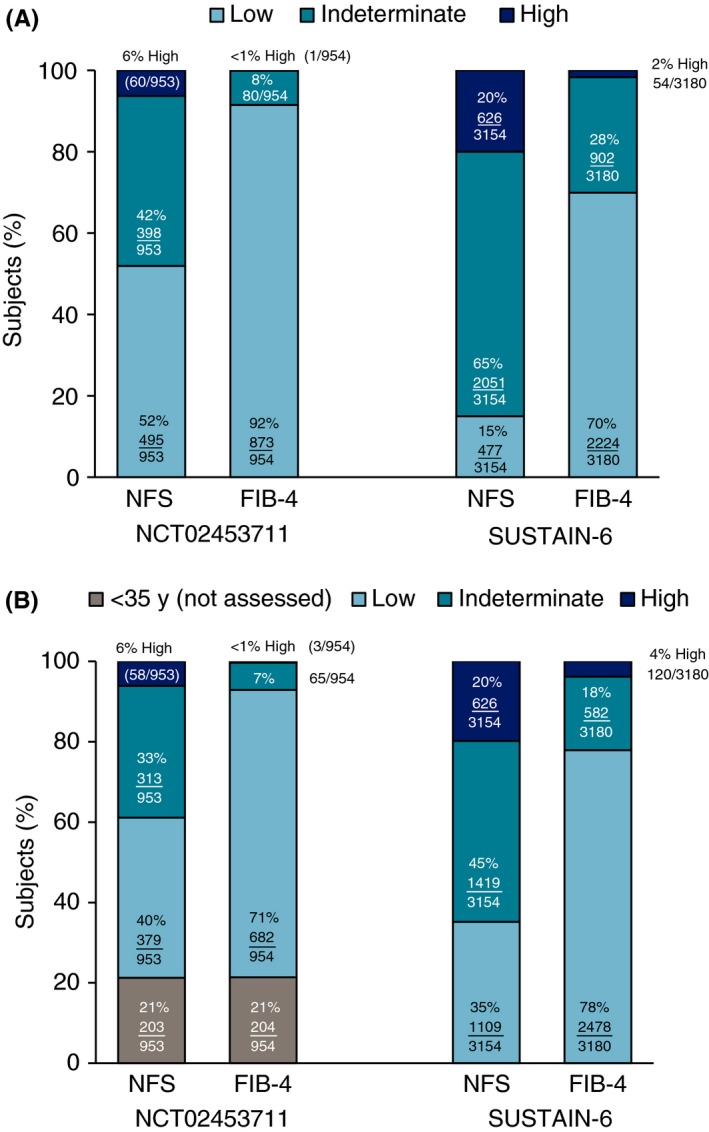
Distribution of baseline fibrosis scores (A) age‐unadjusted; and (B) age‐adjusted. FIB‐4, Fibrosis 4 Index; NFS, non‐alcoholic fatty liver disease Fibrosis Score

There were marked differences in the distribution of both the NAFLD Fibrosis Score and Fibrosis 4 Index markers between the two trials. Median scores for both markers were higher among subjects in the cardiovascular outcomes trial than in the weight management trial (Table 1). Using the global thresholds described above, higher proportions of subjects in the cardiovascular outcomes trial had high or indeterminate NAFLD Fibrosis Score and Fibrosis 4 Index values, and fewer had a low value than in the weight management trial (Figure 1A). The use of age‐stratified marker thresholds made little difference to the proportions with high values of either marker in the weight management trial (Figure 1B). Notably, 21% of weight management subjects were excluded from categorisation as they were under 36 years of age. In the cardiovascular outcomes trial, in which none of the subjects was young enough to be excluded in this way, age‐stratified thresholds resulted in the re‐classification of 31% of the “indeterminate” NAFLD Fibrosis Score stratum (632/2051) as “low”, while there was no change to the number in the high stratum. For the Fibrosis 4 Index, age‐stratified thresholds increased the proportions with both a low and high value while reducing the size of the indeterminate stratum.

### 3.2 Changes in ALT

**Figure 2:**
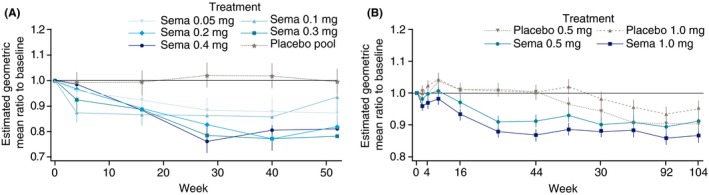
Estimated (mixed model for repeated measurements) mean ALT changes from baseline by treatment group and study visit for individuals with high baseline ALT in (A) weight management trial NCT02453711 and (B) cardiovascular outcomes trial SUSTAIN‐6. ALT, alanine aminotransferase

Figure 2 shows model‐estimated changes from baseline in ALT by treatment visit for individuals with elevated baseline ALT in the weight management (Figure 2A) and cardiovascular outcomes trials (Figure 2B). Dose‐dependent decreases in ALT were observed in both trials, with maximal declines occurring by approximately week 28 and remaining stable thereafter until the end of treatment at week 52 or week 104, despite continuing weight loss. The reduction in ALT was larger in the weight management trial.

**Figure 3:**
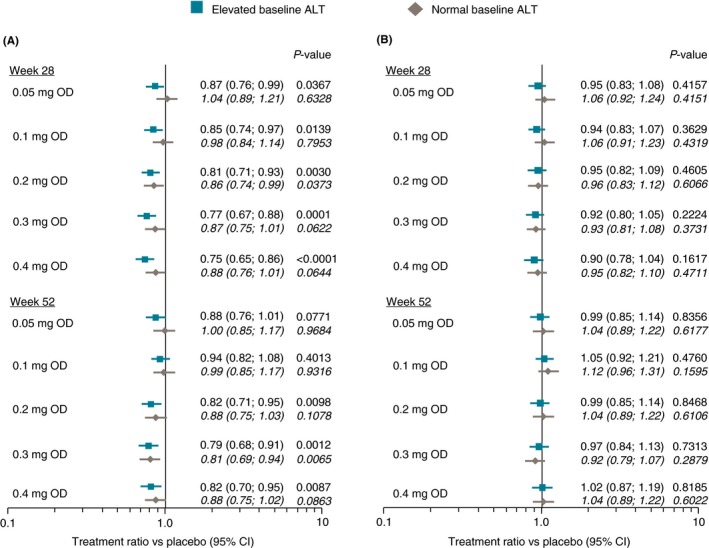
Treatment vs placebo ratios for change in ALT from baseline to weeks 28 or 52 in weight management trial NCT02453711 (A) unadjusted for change in body weight; and (B) adjusted for change in body weight. ALT, alanine aminotransferase; CI, confidence interval; OD, once daily

Treatment ratios vs placebo for ALT change at weeks 28 or 52 (end of treatment) in the weight management trial are shown in Figure 3. In analyses unadjusted for change in body weight (Figure 3A), significant ALT reductions vs placebo of up to 25% were observed in the high baseline ALT group for all semaglutide doses above 0.1 mg/day. Consistent with the absolute declines in ALT observed at each visit, there was no additional decrease in treatment ratios at week 52 compared with week 28. Numerically lower reductions were seen in the group with normal baseline ALT that generally failed to reach statistical significance. After adjustment for weight change, no treatment ratio was statistically significant and all ratios clustered around 1.0 (Figure 3B).

There was no clear influence of sex or age on weight‐unadjusted ALT treatment ratios (Figures S1 and S2), with the caveat that lack of power makes it impossible to draw statistical conclusions.

**Figure 4:**
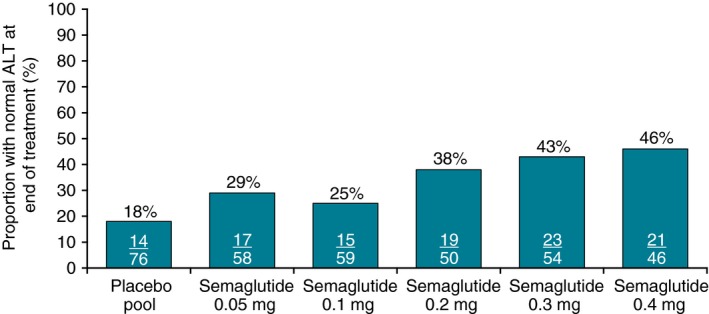
Normalisation of ALT at week 52 among subjects with elevated baseline ALT in weight management trial NCT02453711. ALT, alanine aminotransferase

The treatment‐related reductions in ALT in the weight management trial resulted in normalisation of ALT at week 52 in 25%‐46% of subjects with elevated baseline ALT who received semaglutide, vs 18% who received placebo (Figure 4).

Treatment ratios for ALT change vs placebo at weeks 30, 56 and 104 in the cardiovascular outcomes trial are shown in Figure S3A (unadjusted for weight change) and S3B (adjusted for weight change). At the higher semaglutide dose of 1.0 mg/week, a statistically significant reduction of 9% vs placebo was seen in the high baseline ALT subgroup at week 104. A reduction was also seen for the lower 0.5 mg/week dose at week 30 but this was not sustained to week 56. As with the weight management trial, statistical significance was lost and all ratios clustered around 1.0 after adjustment for change in body weight.

### 3.3 Changes in hsCRP

**Figure 5:**
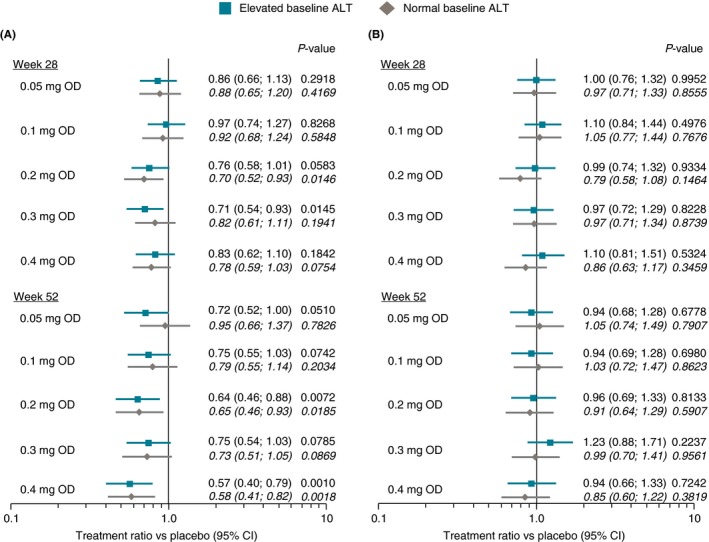
Treatment vs placebo ratios for high‐sensitivity C‐reactive protein change from baseline to weeks 28 or 52 in weight management trial NCT02453711 (A) unadjusted for change in body weight; and (B) adjusted for change in body weight. ALT, alanine aminotransferase; CI, confidence interval; OD, once daily

Treatment ratios vs placebo for the change in hsCRP level from baseline to weeks 28 or 52 in the weight management trial are shown in Figure 5. In contrast to ALT, reductions in hsCRP were comparable between the elevated baseline ALT and normal baseline ALT subgroups, and numerically larger at week 52 than at week 28 (Figure 5A). By week 52, reductions in hsCRP of up to 43% vs placebo were seen for all semaglutide treatment groups that were either statistically significant at the 5% level or close to significance. As with the ALT analysis, statistical significance was lost and all treatment ratios clustered around 1.0 when adjusted for change in body weight (Figure 5B).

There was no apparent influence of sex or age on hsCRP reductions vs placebo (Figures S4 and S5), and there was no quantitative correlation between week 52 changes in hsCRP and ALT. Across the five active treatment groups and placebo, Pearson correlation coefficients ranged between 0.076 and 0.188.

### 3.4 Changes in metabolic syndrome

**Figure 6:**
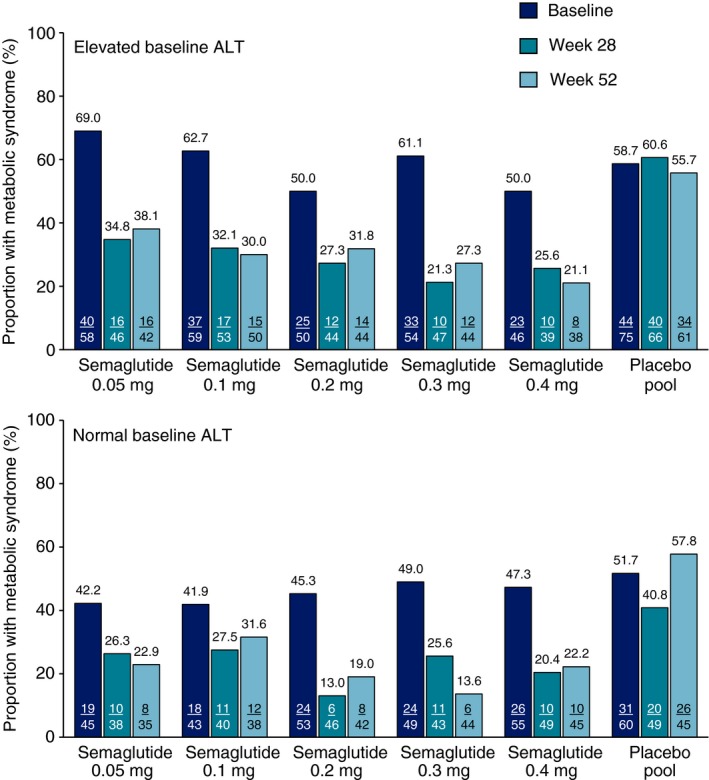
Proportion of subjects in weight management trial NCT02453711 with metabolic syndrome at baseline, week 28 and week 52 of treatment with once‐daily semaglutide or placebo (observed data). ALT, alanine aminotransferase. Metabolic syndrome was defined as three or more of: waist circumference ≥89 cm (women) or ≥102 cm (men); triglycerides ≥1.7 mmol/L; high‐density lipoprotein‐cholesterol <1.3 mmol/L (women) or <1.04 mmol/L (men); systolic blood pressure ≥130 mmHg and diastolic blood pressure ≥85 mmHg; fasting plasma glucose ≥5.6 mm/L

The proportions of subjects in the weight management trial with metabolic syndrome at baseline, week 28 and week 52 are shown by treatment group in Figure 6. Among those who received semaglutide 0.4 mg, metabolic syndrome was reduced from 50.0% at baseline to 25.6% at week 28 in the high baseline ALT subgroup, with a similar decline in the normal ALT subgroup. This decline was broadly similar across semaglutide dosing groups and stable between week 28 and week 52. Thus, among subjects treated with semaglutide, the proportion with metabolic syndrome was approximately halved during the trial compared with the baseline proportion. In contrast, the proportion with metabolic syndrome in the pooled placebo group remained unchanged between baseline and week 52 for both the elevated and normal ALT subgroups.

## 4 DISCUSSION

In two large clinical trials, in which semaglutide was used to treat different patient groups for which NAFLD is a known comorbidity, there were clear dose‐dependent reductions in both ALT and hsCRP.

A high proportion of subjects in both trials had elevated ALT levels at baseline. The extent to which ALT was elevated was similar across these two different patient groups, but the proportion with an elevated level was higher in the obesity group (52%) than in the group with diabetes and high cardiovascular risk (41%). There was also a high proportion of subjects in both trials who had metabolic syndrome at baseline, and 65% of subjects in the obesity group had elevated baseline hsCRP suggestive of systemic inflammation.

Elevated baseline ALT was significantly reduced on semaglutide treatment in both trials, and these reductions were broadly dose proportional and greater at the higher dosing in the weight management trial than in the cardiovascular outcomes trial. In both trials, maximal ALT reductions were typically seen after 28‐30 weeks of treatment, and remained constant thereafter until end of treatment at week 52 or 104. In the weight management trial, these reductions resulted in dose‐dependent normalisation of elevated baseline ALT in up to 46% of those who received the highest semaglutide dose of 0.4 mg/day, vs 18% on placebo.

ALT reduction appeared to be associated with weight reduction, suggested by the loss of a significant treatment effect and the clustering of treatment ratios around 1.0 in the exploratory analysis adjusting for change in body weight. This is consistent with the association of excess adiposity to the pathogenesis of NAFLD,^35^, ^36^ and with previous data showing a significant ALT reduction in patients with type 2 diabetes given 26 weeks of liraglutide 1.8 mg/day that was similarly attenuated after adjustment for weight change.^22^ However, weight loss is not the only mechanism by which the glucagon‐like peptide‐1 receptor agonists may exert pleiotropic beneficial effects on metabolism, steatosis, cardiovascular risk and inflammation.^16^, ^37^, ^38^, ^39^, ^40^, ^41^, ^42^ Thus, while these results indicate an association between weight loss and ALT change, neither causality nor sole agency can be established, and further research will be needed to evaluate other potential contributors or whether weight loss achieved by other means yields the same ALT reductions seen here.

Semaglutide treatment similarly showed an effect on hsCRP in the weight management trial where this parameter was assessed, showing significant and broadly dose‐dependent reductions that were also linked with weight reduction. Unlike ALT, hsCRP reductions appeared to continue beyond 30 weeks. As hsCRP was not assessed in the cardiovascular outcomes trial, it is not possible to estimate the reductions that would be achieved without the dietary and lifestyle interventions undertaken in the weight management study.

It was also of note that the prevalence of metabolic syndrome, a strong predictor of NAFLD/NASH,^43^, ^44^ decreased substantially over 52 weeks of semaglutide treatment in the weight management trial where this was assessed longitudinally.

The principal limitation of these analyses was that neither trial enrolled subjects with confirmed NASH and histology data were not available. Thus, while the baseline characteristics from these trials are consistent with the presence of NAFLD/NASH in many or most subjects, this inference is untestable within these datasets and so the empirical results of the analysis cannot be directly linked to the presence or severity of fatty liver disease.

Within the constraints of this limitation, data for ALT and/or hsCRP reductions from interventional studies in confirmed NASH are of interest. ALT has been demonstrated to decline in patients with treatment‐related improvement or resolution of histological NASH in studies of obeticholic acid,^45^ elafibranor^46^ and liraglutide,^23^ and also shown to decline in association with liver fat reductions among patients with type 2 diabetes and NAFLD treated with the sodium‐glucose cotransporter 2‐inhibitor empagliflozin.^47^ While correlated outcomes do not always indicate a causative association, several studies have also observed a predictive association between changes in ALT and subsequent NAFLD/NASH‐related outcomes. In the GOLDEN‐505 trial of elafibranor for NASH, higher baseline ALT was associated with more active histological disease and declines during the trial were associated with histological improvement, with NASH resolution associated with the strongest time‐dependent ALT reductions.^48^ In the FLINT trial of obeticholic acid for NASH, a week 24 ALT reduction ≥17 U/L independently predicted week 72 histological response.^49^ Furthermore, in the PIVENS trial, normalisation of elevated ALT at week 24 (a ≥30% reduction from baseline resulting in a level ≤40 U/L) without subsequent relapse was strongly associated with histological improvement of NASH activity at week 96 among subjects receiving either vitamin E or placebo.^27^ Finally, normalisation of high baseline ALT has independently predicted fibrosis improvement in patients with NASH given 1 year of lifestyle intervention.^50^

Elevated hsCRP is predictive of both type 2 diabetes^51^ and cardiovascular risk,^52^ and is a risk factor for steatosis.^26^, ^53^, ^54^, ^55^, ^56^, ^57^, ^58^ However, its association with NASH is less clearly defined: some studies show no association between hsCRP and the severity of NAFLD,^55^, ^56^ while others found that hsCRP can discriminate between steatosis and NASH, particularly more severe NASH,^26^, ^57^, ^58^ and is associated with underlying fibrosis.^26^, ^57^ Thus, ALT and hsCRP may reflect different aspects of the pathogenic process, and their mutual reduction may represent separate treatment effects.

A disconnection between hsCRP, ALT and histological benefit was observed in the recent phase 2 CENTAUR trial of the CCR2/CCR5 antagonist cenicriviroc for treatment of NASH. After 1 year of treatment, significant reductions were observed vs placebo in both hsCRP and biopsy‐assessed fibrosis, but there was no significant treatment effect on either biopsy‐assessed NASH or ALT.^59^ Thus, although these limited observations should be interpreted with caution given the absence of mechanistic data, it is possible that ALT and hsCRP differ in their strengths of association with active steatohepatitis and fibrotic activity, with ALT potentially more closely linked to the former and hsCRP to the latter. The concomitant reduction of both by semaglutide may imply a beneficial effect on liver necroinflammation for both NASH activity and fibrosis, though this will require histological confirmation in a population with confirmed NASH.

In conclusion, semaglutide treatment significantly reduced elevated ALT and hsCRP in individuals at high risk of NAFLD. These reductions were greatest at the higher doses of semaglutide used and were linked to the degree of weight loss. The ability of glucagon‐like peptide‐1 receptor agonists to reduce weight and lower ALT levels implies a potential role for these compounds in NAFLD/NASH treatment, and histological data are awaited from an ongoing phase 2 trial of semaglutide in biopsy‐proven NASH (NCT02970942).

## AUTHORSHIP

*Guarantor of the article:* Philip Newsome.

*Author contributions:* The sponsor, Novo Nordisk, contributed to the designs of both studies; John P H Wilding also contributed to the design of the study, evaluating the efficacy and safety of semaglutide in comparison with liraglutide and placebo in promoting weight loss (NCT02453711). Data were gathered by the site investigators, and the sponsor performed site monitoring, data collection and data analysis for both studies. All authors participated in interpretation of the data, and the drafting and revision of the manuscript. All authors reviewed and approved the final, submitted version.
