# Supplementary material for: Effect of semaglutide on liver enzymes and markers of inflammation in subjects with type 2 diabetes and/or obesity
Source: Aliment Pharmacol Ther. 2019 Jun 10;50(2):193–203. doi: 10.1111/apt.15316 (PMC6617813; doi:10.1111/apt.15316)
Supplement: Supplementary file 1 [file APT-50-193-s001.pdf]

Supporting information

Effect of semaglutide on liver enzymes and markers of inflammation in subjects with type 2 diabetes and/or obesity

Philip Newsome, et al.

Contents

Figure S1. MMRM-estimated treatment vs. placebo ratios for change in ALT from baseline to weeks 28 or 52 in weight management trial NCT02453711, by baseline ALT subgroup and sex. 3

Figure S2. MMRM-estimated treatment vs. placebo ratios for change in ALT from baseline to weeks 28 or 52 in weight management trial NCT02453711, by baseline ALT subgroup and age relative to the median (47 years). 4

Figure S3. MMRM-estimated treatment vs. placebo ratios for change in ALT from baseline to weeks 30, 56 or 104 in cardiovascular outcomes trial SUSTAIN-6 (A) unadjusted for change in body weight; and (B) adjusted for change in body weight. 5

Figure S4. MMRM-estimated treatment vs. placebo ratios for change in hsCRP from baseline to weeks 28 or 52 in weight management trial NCT02453711, by baseline ALT subgroup and sex. 6

Figure S5. MMRM-estimated treatment vs. placebo ratios for change in hsCRP from baseline to weeks 28 or 52 in weight management trial NCT02453711, by baseline ALT subgroup and age relative to the median (47 years). 7

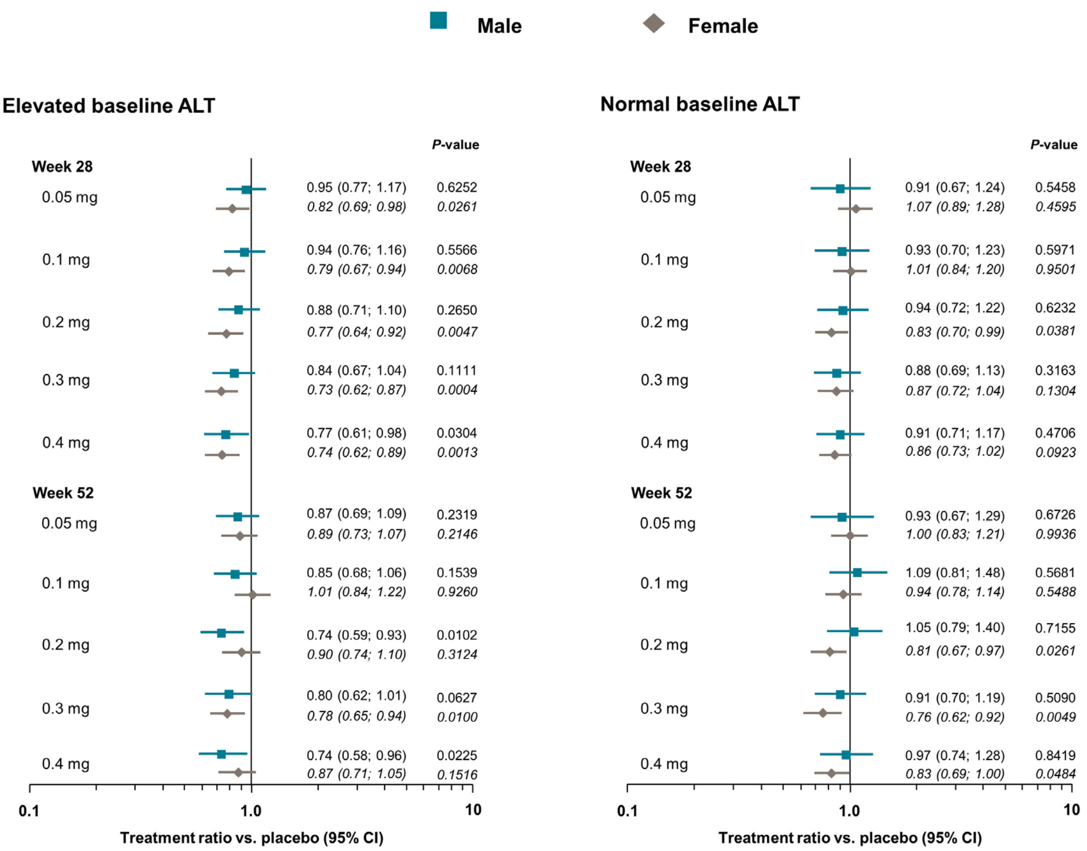

Figure S1. MMRM-estimated treatment vs. placebo ratios for change in ALT from baseline to weeks 28 or 52 in weight management trial NCT02453711, by baseline ALT subgroup and sex. ALT, alanine aminotransferase; CI, confidence interval;
MMRM, mixed model for repeated measurements.

Elevated ALT was defined as >30 IU/mL in men or >19 IU/mL in women.

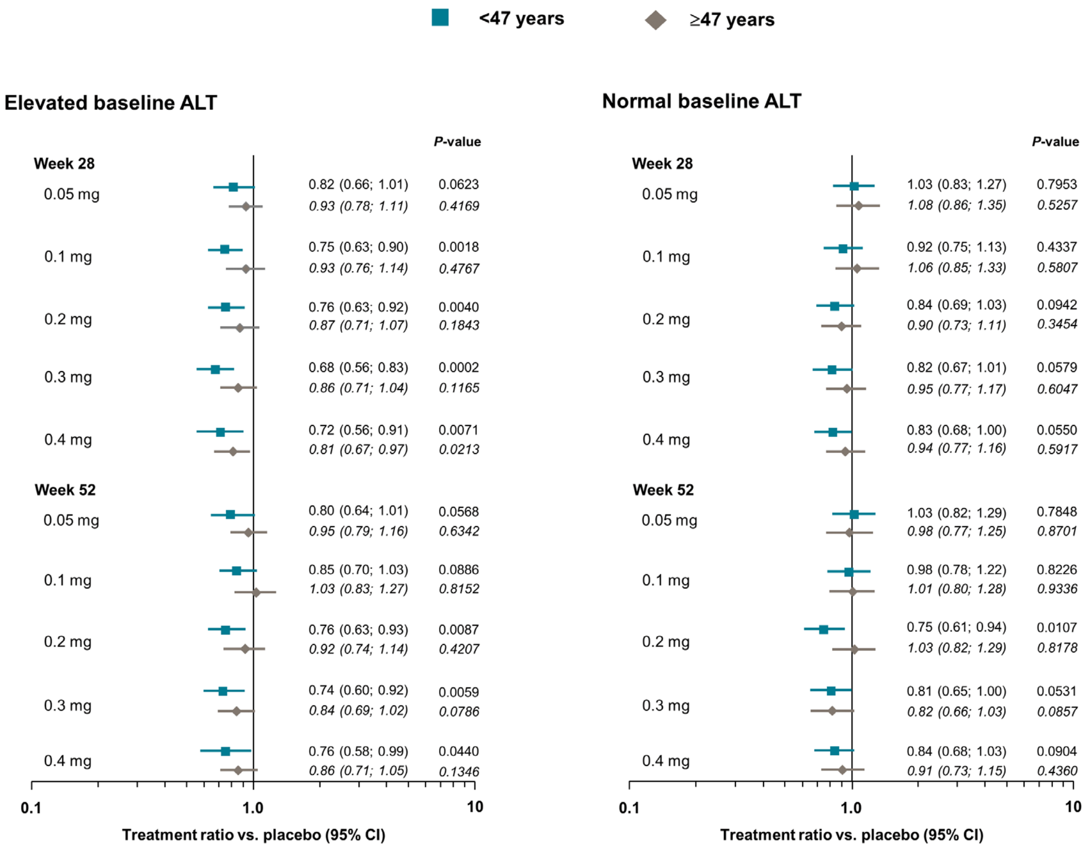

Figure S2. MMRM-estimated treatment vs. placebo ratios for change in ALT from baseline to weeks 28 or 52 in weight management trial NCT02453711, by baseline ALT subgroup and age relative to the median (47 years).
ALT, alanine aminotransferase; CI, confidence interval;
MMRM, mixed model for repeated measurements.

Elevated ALT was defined as >30 IU/mL in men or >19 IU/mL in women.

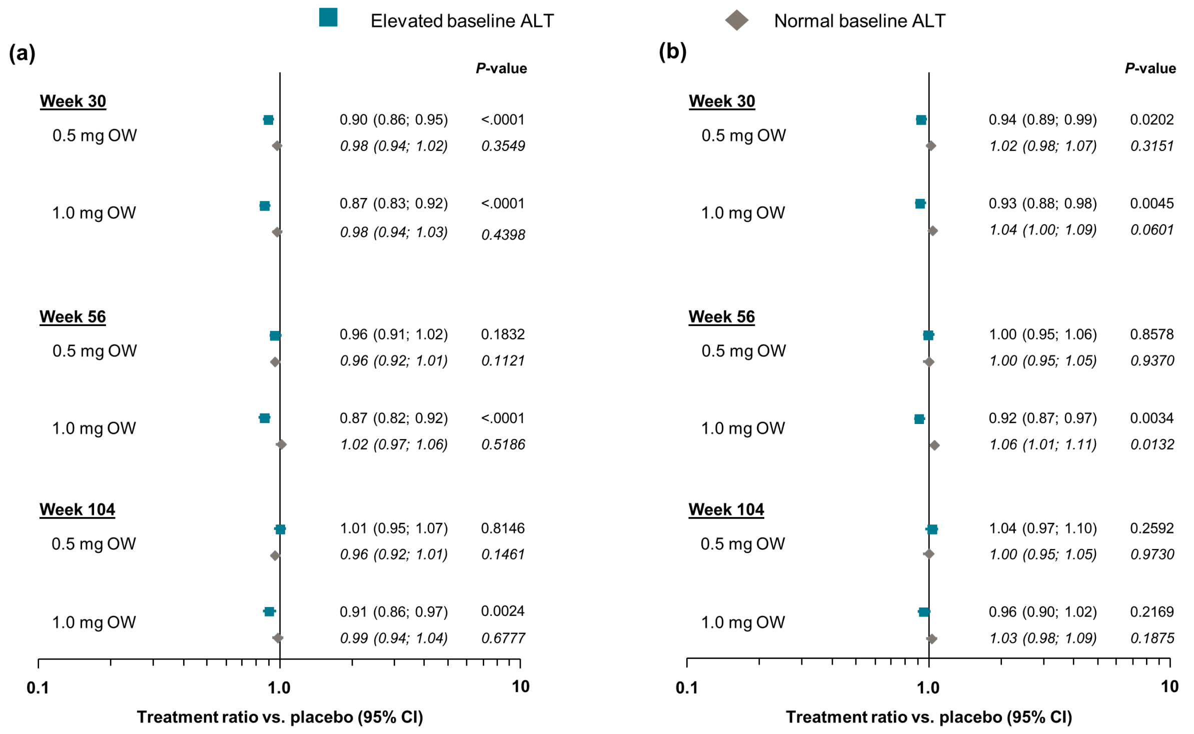

Figure S3. MMRM-estimated treatment vs. placebo ratios for change in ALT from baseline to weeks 30, 56 or 104 in cardiovascular outcomes trial SUSTAIN-6 (A) unadjusted for change in body weight; and (B) adjusted for change in body weight.
ALT, alanine aminotransferase; CI, confidence interval;
MMRM, mixed model for repeated measurements; OW, once weekly.

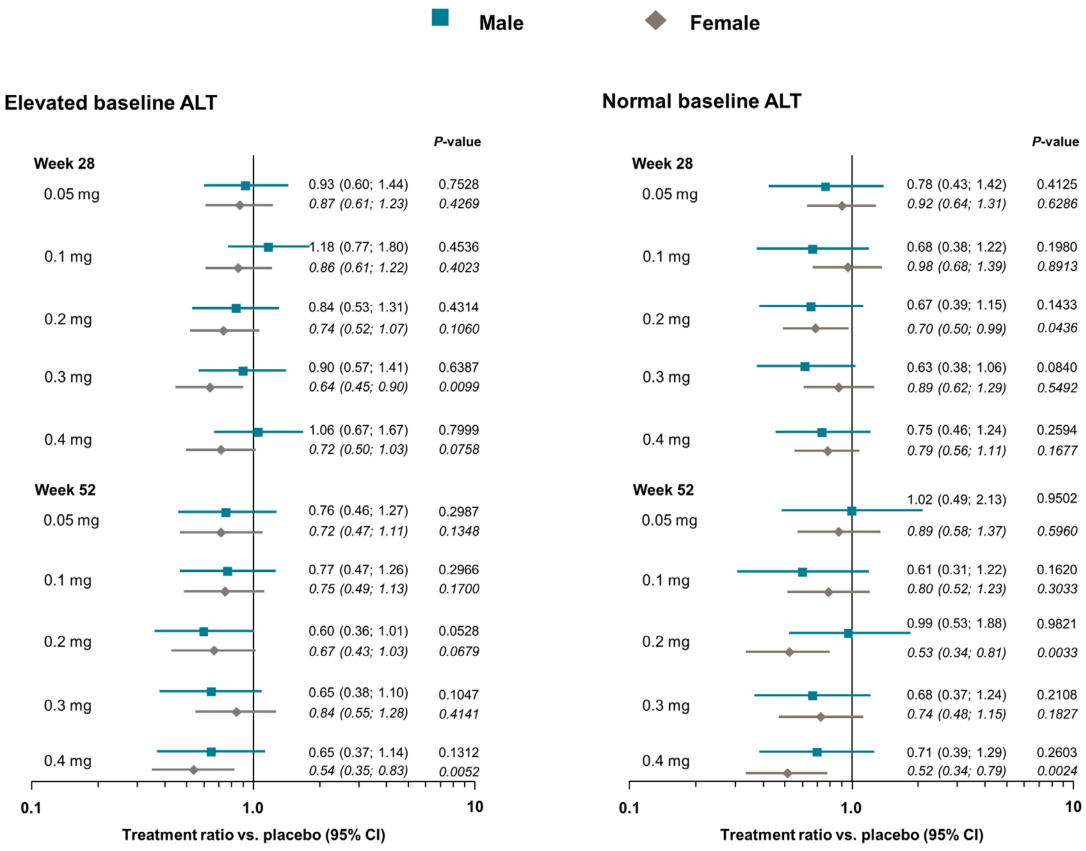

Figure S4. MMRM-estimated treatment vs. placebo ratios for change in hsCRP from baseline to weeks 28 or 52 in weight management trial NCT02453711, by baseline ALT subgroup and sex. ALT, alanine aminotransferase; CI, confidence interval; hsCRP, high-sensitivity C-reactive protein; MMRM, mixed model for repeated measurements.

Elevated ALT was defined as >30 IU/mL in men or >19 IU/mL in women.

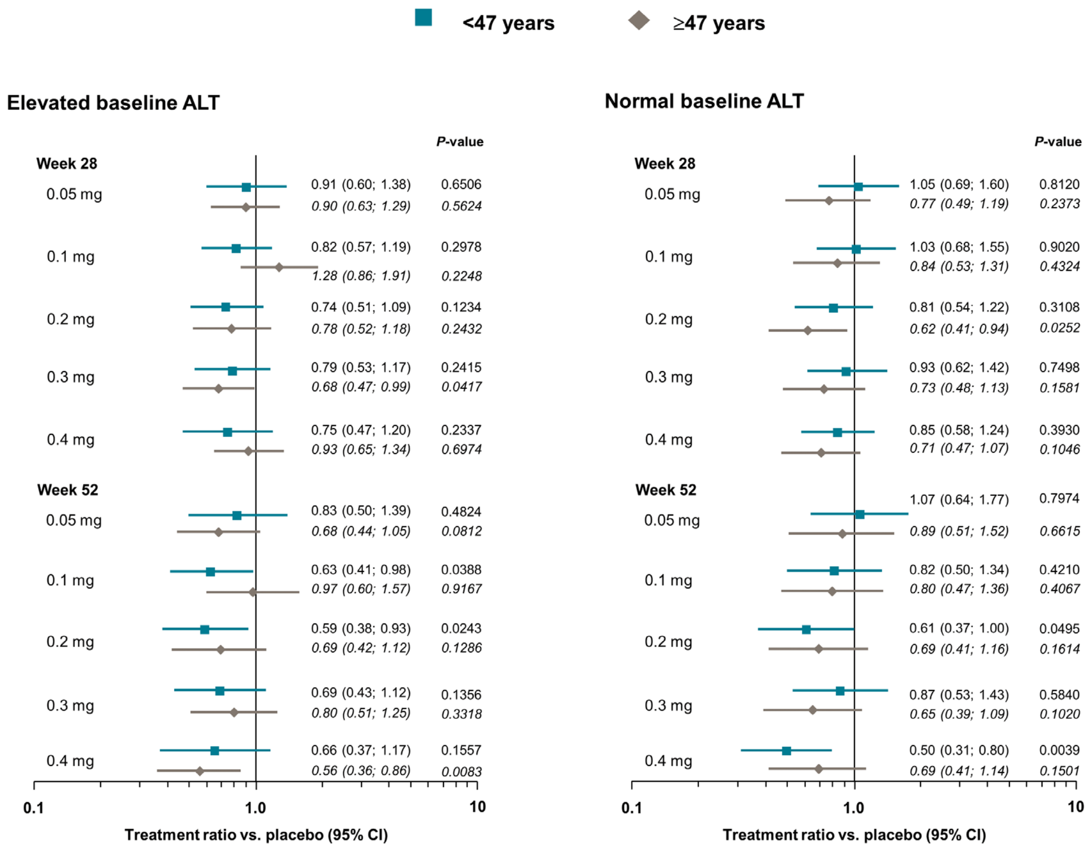

Figure S5. MMRM-estimated treatment vs. placebo ratios for change in hsCRP from baseline to weeks 28 or 52 in weight management trial NCT02453711, by baseline ALT subgroup and age relative to the median (47 years).
ALT, alanine aminotransferase; CI, confidence interval; hsCRP, high-sensitivity C-reactive protein; MMRM, mixed model for repeated measurements.

Elevated ALT was defined as >30 IU/mL in men or >19 IU/mL in women.
